# Amniotic Fluid Embolism Coagulopathy Guided by the Point-of-Care Quantra QStat® Hemostasis System: A Case Report

**DOI:** 10.7759/cureus.55387

**Published:** 2024-03-02

**Authors:** Eugénie Fradin, Olivier Belin, Didier Bonnet, Isabelle Caron, Thomas Brungs

**Affiliations:** 1 Department of Anesthesiology and Reanimation, University Hospital Orleans, Orleans, FRA; 2 Department of Anesthesiology and Reanimation, University Hospital of Orleans, Orleans, FRA; 3 Department of Obstetrics and Gynecology/Anesthesiology, University Hospital of Orleans, Orleans, FRA; 4 Department of Hematology/Laboratories, University Hospital of Orleans, Orleans, FRA

**Keywords:** disseminated intravascular coagulation, postpartum hemorrhage, point-of-care viscoelastic testing, acute obstetric coagulopathy, amniotic fluid embolism, anesthesia

## Abstract

Amniotic fluid embolism (AFE) is a rare pregnancy complication associated with high maternal mortality that occurs during labor or in the early postpartum period. The diagnosis of AFE is challenging because signs and symptoms are common to other obstetric complications. Early identification and management of profound coagulopathy associated with AFE is essential to improve patient survival. We present a case of a 31-year-old woman with placenta previa and clinical suspicion of AFE after cesarean section. Immediately after delivery, the parturient presented hypotension, hypoxia, coagulopathy, and severe postpartum hemorrhage. We hereby discuss the role of the most recently developed point-of-care viscoelastic testing device, the Quantra QStat^®^ system (Stago Group Company; HemoSonics LLC, Durham, NC), for early detection of acute obstetric coagulopathy and guided hemostatic treatment.

## Introduction

Postpartum hemorrhage (PPH) is the leading direct cause of maternal death worldwide, accounting for approximately 70,000 deaths each year [1]. A rare cause of PPH is amniotic fluid embolism (AFE), a complication that occurs during labor or in the early postpartum period with high maternal mortality [2]. In France, AFE is the third most common cause of maternal mortality [3]. From 2013 to 2015, AFE was responsible for the death of 28 women during the year following childbirth, with a mortality rate of 1.2 per 100,000 live births. The reported incidences of AFE range from 0.4 to 4.4 per 100,000 births in Europe [4]. Although the pathophysiology of AFE is not completely understood, disruption of maternal-fetal interface is thought to lead to abnormal activation of proinflammatory mediators and a cascade of deleterious events [5]. The clinical signs of AFE include collapse, respiratory distress, hypoxia, acute hypotension, cardiovascular arrest, disseminated intravascular coagulation (DIC), and neurological problems. The diagnosis of AFE is challenging because signs and symptoms are common to other obstetric complications. Further, a gold-standard diagnostic test is not available, and several diagnostic criteria have been proposed [5,6]. Older maternal age, placenta previa, placental abruption, cesarean section, and instrumental vaginal delivery have been identified as risk factors [4]. Recommendations for the initial care of AFE comprise cardiopulmonary resuscitation, massive transfusion protocol (to control bleeding and correct coagulopathy), and management of pulmonary hypertension and right ventricular failure [2,7,8].

Standard blood coagulation tests such as prothrombin time (PT), activated partial thromboplastin time (aPTT), platelet count, and fibrinogen level are traditionally used to evaluate the hemostatic status of obstetric patients. However, in the context of acute obstetric coagulopathy (AOC) and severe hemorrhage, blood transfusions are usually required before the results of the blood tests are available. Point-of-care viscoelastic testing (POCVT) of whole blood samples provides timely evaluation of the patient’s coagulation status at the bedside, allowing for better treatment of coagulopathy during PPH [9,10]. The use of thromboelastography (TEG®; Haemonetics Corp., Braintree, MA) and rotational thromboelastometry (ROTEM®; Werfen, Barcelona, Spain) POCVT devices in the management of AFE was previously reported [11-14].

We present a case of suspected AFE managed with the assistance of the most recently developed POCVT device - the Quantra QStat® system (Stago Group Company; HemoSonics LLC, Durham, NC). The Quantra QStat® system is based on a unique ultrasound technology, the Sonic Estimation of Elasticity via Resonance (SEER) Sonorheometry, which induces shear wave resonance within a whole blood sample. The characteristics of the resonance are analyzed over time to evaluate changes in the clot shear modulus of elasticity [10,15]. The Quantra QStat® system works with the ready-to-use QStat® four-channel cartridge and displays five parameters: clot time (CT), clot stiffness (CS), fibrinogen contribution to clot stiffness (FCS), platelet contribution to clot stiffness (PCS), and clot stability to lysis (CSL) [10]. While CT, CS, and FCS are directly measured, PCS and CSL are calculated. The PCS parameter is the difference between CS and FCS. The CSL parameter is the normalized difference of the CS change after maximum CS in the absence of tranexamic acid (TXA) and the corresponding CS change in the presence of TXA. By measuring changes in CT and CS from initiation of clot formation to fibrinolysis, POCVT devices depict the entire coagulation process.

A parturient underwent a cesarean section at term and presented hypotension, hypoxia, and severe PPH immediately after delivery. Measurements with the POCVT Quantra QStat® system allowed for early detection of coagulopathy and informed blood resuscitation procedures. POCVT may assist clinicians in promptly and efficiently managing AOC associated with AFE, potentially improving maternal outcomes.

## Case presentation

A 31-year-old woman, gravida 4 para 2, was admitted to the operating room of the maternity unit at 37 weeks of gestation, for a scheduled cesarean section for placenta previa. The healthy parturient had no significant past medical history. Spinal anesthesia was performed at 2:53 p.m. with sufentanil, bupivacaine, and a low dose of morphine.

At 3:10 p.m., immediately after the birth of the baby and the delivery of the placenta, the patient showed signs of tonic-clonic seizures, loss of consciousness, tachycardia, hypotension, and hypoxia. The patient developed hemorrhage with an estimated blood loss of 1.1 L. Clinical suspicion of AFE was based on the primary symptoms. Noradrenaline was administered to restore blood pressure. Early uterotonic medication was initiated with oxytocin quickly followed by sulprostone. An orotracheal intubation was performed for ventilation and fluid resuscitation was administered. The surgical wound was closed, and 1 g of TXA was infused intravenously to minimize blood loss.

The patient’s hemostatic status was monitored in real time using the Quantra QStat® system. The first results, obtained 45 minutes after delivery, revealed a normal CT of 151 s (normal range 113-165 s) and low CS of 8.4 hPa (normal range 16.0-33.2 hPa). Both functional FCS of 0.4 hPa (normal range 1.9-3.7 hPa) and PCS of 8.0 hPa (normal range 14.1-29.8 hPa) were very low. Blood samples were also collected and sent for standard blood testing. Retrospectively, the results showed hemoglobin (Hb) levels of 8.1 g/dL, PT of 63%, aPTT ratio of 2.21, platelet count of 139 × 10^9^ cells/L, Clauss fibrinogen level of 1.1 g/L, factor V activity of 55%, and D-dimer level >24,000 ng/mL. The findings were consistent with the DIC associated with AFE. Venous blood gas analysis revealed a low pH of 7.26 and lactate levels of 1.9 mmol/L. The PPH management protocol was activated. The patient received one unit of red blood cells (RBCs) and 1.5 g of fibrinogen concentrate. The hemorrhage continued, and approximately 30 minutes after transfusion initiation, an intrauterine Bakri balloon was applied. The patient received two additional units of RBC, one unit of fresh frozen plasma (FFP), 3.0 g of fibrinogen concentrate, and albumin.

**Figure 1:**
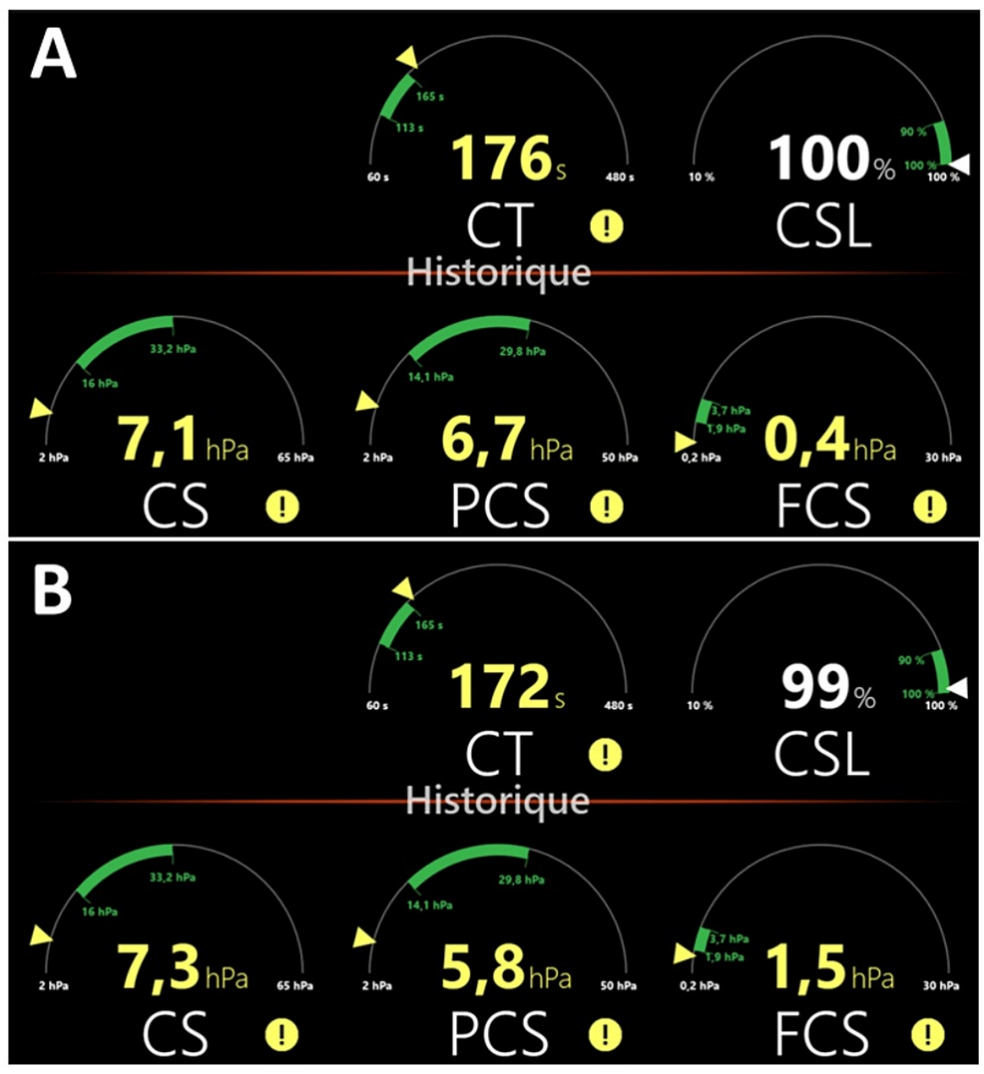
Viscoelastic hemostatic status of the patient evaluated using the Quantra QStat® system. Whole blood measurements were performed (A) two hours and (B) seven hours after placenta delivery. Parameters obtained with Quantra QStat® cartridge include clot time (CT), clot stiffness (CS), fibrinogen contribution to clot stiffness (FCS), platelet contribution to clot stiffness (PCS), and clot stability to lysis (CSL). Normal reference ranges are indicated by the green areas.

Two hours after delivery, vaginal bleeding persisted. The second Quantra QStat® test revealed normal CSL of 100% (normal range 90%-100%), prolonged CT, and a further decrease in CS and PCS (7.1 and 6.7 hPa, respectively). The FCS level of 0.4 hPa remained very low (Figure 1A). The results informed the subsequent blood product transfusion. The patient received one unit of RBC, five units of FFP, 4.5 g of fibrinogen concentrate, and one unit of platelet concentrate and calcium chloride. Three hours after delivery, bleeding was still observed. The third Quantra QStat® test confirmed ongoing coagulopathy (CT of 200 s, CS of 7.0 hPa, FCS of 0.4 hPa, and PCS of 6.6 hPa). Two additional units of RBCs, four units of FFP, and 4.5 g of fibrinogen concentrate were transfused. When the Bakri balloon was removed one hour later, bleeding increased and clots were visible. To manage the resistant obstetric hemorrhage and coagulopathy, a more aggressive approach was taken, and an emergency laparoscopic hysterectomy was initiated. Shortly after, an estimated additional blood loss of 1.7 L was observed, with the patient presenting signs of hemorrhagic shock. The patient received four units of RBC, four units of FFP, 4.5 g of fibrinogen concentrate, 7.0 mg of recombinant activated factor VII (rFVIIa), calcium chloride, and albumin. The fourth Quantra QStat® test revealed hemostatic impairment, with a further decline in CS parameters (CS of 2.3 hPa, FCS of 0.2 hPa, and PCS of 2.1 hPa). Retrospectively, standard blood tests showed a low Hb of 5.9 g/dL, high aPTT ratio of 2.47, low Clauss fibrinogen level of 0.75 g/L, and low platelet count of 64 × 10^9^ cells/L. Antibiotic prophylaxis and volume management were performed. One hour after surgery initiation, a further estimated blood loss of 1.2 L was observed. From the start of the cesarean section to this moment, the total estimated blood loss was 5.3 L. The patient received five additional units of RBC, two units of FFP, 4.5 g of fibrinogen concentrate, and one unit of platelet concentrate, after which the incisions were closed.

One hour after surgery termination, the fifth Quantra QStat® test revealed improvement in CT and CS parameters (Figure 1B). At 11:21 p.m., the patient was transferred from the operating room to the intensive care unit without signs of active bleeding. At 11:32 p.m., standard blood tests revealed improvement of Hb, PT, aPTT ratio, and Clauss fibrinogen levels (9.4 g/dL, 148%, and 1.21 and 2.63 g/L, respectively) and a low platelet count (63 × 10^9^ cells/L).

One day after delivery, the patient was successfully extubated, and oxygen therapy was initiated. Two days after delivery, the patient was transferred to the ward where clinical status continued to improve. Standard blood tests revealed Hb levels of 9.5 g/dL and a normal platelet count of 85 × 10^9^ cells/L. The creatinine level was normal, and no acute kidney injury was observed.

Mother and baby were discharged from the obstetrics department five days after delivery in stable condition.

A sample of the maternal blood, collected 45 minutes after delivery, was stored and later sent to the Expert Laboratory of Lyon for quantification of serum tryptase and insulin-like growth factor binding protein-1 (IGFBP-1).

## Discussion

We present a parturient who underwent elective cesarean section for placenta previa. Immediately after delivery, the patient developed sudden hypotension and hypoxia. Quantra QStat® testing revealed profound coagulopathy characterized by very low levels of functional fibrinogen (FCS of 0.4 hPa). Retrospectively, blood samples collected after the onset of symptoms revealed a low Clauss fibrinogen level of 1.1 g/L, low factor V activity of 55%, and elevated D-dimer level. The patient developed severe PPH, attributed to coagulopathy associated with AFE, leading to intrauterine hemorrhage and subsequent atony. After several unsuccessful attempts to control bleeding and correct coagulopathy, the clinicians performed a laparoscopic hysterectomy as a lifesaving procedure. The total estimated blood loss was 5.3 L and blood resuscitation was guided by POCVT. The patient received a total of 15 units of RBC, 20 units of FFP, two units of platelet concentrate, 24 g of fibrinogen concentrate, and 7 mg of rFVIIa. Care management lasted for nine hours during which a total of five Quantra QStat® tests were performed.

The 2022 guidelines for the management of severe peri-operative bleeding from the European Society of Anesthesiology and Intensive Care (ESAIC) highlight the need to increase risk awareness and early recognition of severe PPH in patients undergoing obstetric surgery [16]. The ESAIC guidelines recommend the development of protocol-based interventions and patient blood management programs to promote early access to blood products as well as to reduce the use of blood transfusions (grade of recommendation: 2B). At our department, we have developed a Quantra QStat®-guided intervention protocol for PPH management. According to our protocol, to assist medical teams and guide blood resuscitation, Quantra QStat® testing must be performed in all cases of PPH (blood loss >1 L or >500 mL, abnormal hemostasis, rapid blood loss, and/or signs of hypovolemic shock) together with complete blood count analysis.

We present a case characterized by early recognition of PPH (initial blood loss of 1.1 L), followed by fast administration of the antifibrinolytic agent TXA at a dose of 1 g, as recommended by ESAIC guidelines [16].

A diagnosis of AFE was made based on the Clark diagnostic criteria [6]. The Clark criteria were developed by the Society for Maternal-Fetal Medicine and the AFE Foundation and include the following: (1) sudden cardiorespiratory arrest, or both hypotension and respiratory compromise; (2) documented DIC, calculated using the scoring system modified for pregnancy of the International Society on Thrombosis and Hemostasis (ISTH); (3) clinical onset during labor or within 30 minutes of delivery; and (4) absence of fever. We present a case that met all Clark criteria for AFE with a calculated ISTH DIC score for pregnancy of three (a score ≥ 3 identifies overt DIC).

Diagnosis of AFE would be facilitated by the identification of an unequivocal biomarker for the disease. Although several potential biomarkers have been investigated, no gold-standard diagnostic test is available to date [7]. While tryptase was not detected in the maternal serum, the levels of IGFBP-1 were elevated further supporting a diagnosis of AFE [17].

The suspected underlying cause of PPH was acute coagulopathy associated with AFE. By the Clark diagnostic criteria for AFE, coagulopathy was detected before the loss of sufficient blood to account for dilutional or shock-related consumptive coagulopathy [6]. Mechanistically, it is proposed that the maternal systemic inflammatory response observed in AFE activates the coagulation extrinsic pathway and results in the consumption of coagulation factors [7].

Disseminated intravascular coagulation occurs in 83% of AFE cases and must be promptly corrected by blood transfusion [7]. Recently, de Lloyd et al. studied a large cohort of women with PPH and identified an uncommon severe coagulopathy profile named AOC [18,19]. The AOC profile was characterized by impaired fibrinogen function and increased levels of plasmin-antiplasmin complexes and D-dimer and was associated with cases of placental abruption and AFE. Early assessment of AOC is crucial to anticipate complications, optimize treatments, improve maternal outcomes, and increase survival rates [18]. Therefore, an appropriate use of POCVT may contribute to early recognition and management of AOC [10].

Point-of-care viscoelastic testing devices are emergent technologies that provide efficient and accurate ways of evaluating coagulopathic states [9]. In comparison to standard blood tests, POCVT presents several advantages, including automation, fast turnaround times, and assessment of the contribution of specific blood components for clot formation. The use of TEG® and ROTEM® devices in the context of AFE management was previously reported [11-14]. To our knowledge, this is the first case reported of early management of AFE assisted by the Quantra QStat® system.

Quantra QStat® tests can quickly inform clinicians about a range of coagulation and fibrinolysis defects [10,15]. In this case, abnormal coagulation was present due to severe hypofibrinogenemia (FCS < 1.9 hPa) and moderate thrombocytopenia/thrombopathy (PCS < 14.1 hPa). There was no evidence of hyperfibrinolysis. Standard blood tests confirmed low levels of fibrinogen and platelet counts. Fibrinogen levels less than 2 g/L indicate a high risk of severe PPH [16]. The observed decline in platelet counts seemed to be correlated with increasing blood loss. Transfusions with allogeneic blood products (RBC, FFP, and platelet concentrate) and coagulation factors (fibrinogen concentrate and rFVIIa) were guided by monitoring Quantra QStat® parameters over time. By enabling the selection of the best blood products to be transfused, Quantra QStat® may contribute to individualized care and improve treatment efficiency. Indeed, ESAIC recommends the use of POCVT-guided hemostatic treatment to reduce the need for blood products [16]. Also, Bell et al. emphasize the utility of POCVT in detecting hypofibrinogenemia due to dilution or acute coagulopathy and guiding fibrinogen replacement [9]. Of note, a high correlation between fibrinogen parameters measured with the Quantra QStat® system and standard fibrinogen concentration analysis was previously shown (in a cohort study of healthy third-trimester pregnant women) [20].

The coagulation factor rFVIIa was administered at a late stage of patient management, after intrauterine tamponade and hysterectomy, when PPH was still considered life-threatening. This procedure agrees with ESAIC recommendations [16].

Unlike typical cases of AFE, sudden cardiorespiratory collapse did not occur [7]. Therefore, cardiopulmonary resuscitation and/or extracorporeal membrane oxygenation was not required. Renal compromise is associated with some cases of AFE, but in this case, renal function remained unaffected [11].

## Conclusions

The obstetric complication AFE is rare but potentially fatal and its diagnosis of exclusion is based on clinical presentation. We present a case of AFE with profound coagulopathy, developed after cesarean section for placenta previa, complicated by severe PPH. This case highlights the use of the most recent POCVT device, the Quantra QStat® system, to acquire rapid, reliable, and actionable coagulopathy information during PPH management. Quantra QStat® testing allowed for early recognition of hypofibrinogenemia and guided fibrinogen replacement and blood transfusions. We suggest that POCVT can assist in the early diagnosis of developing obstetric coagulopathies and inform individualized blood product resuscitation. Further, the implementation of patient blood management measures may contribute to reducing maternal death and complications, as well as improving treatment efficiency.
